# Supplementary material for: Vitamin D Deficiency and Its Association with Iron Deficiency in African Children
Source: Nutrients. 2022 Mar 25;14(7):1372. doi: 10.3390/nu14071372 (PMC9002534; doi:10.3390/nu14071372)
Supplement: Supplementary file 1 [file nutrients-14-01372-s001.zip › nutrients-1611388-supplementary.pdf]

Supplementary Materials

Supplementary Methods S1. Biochemical assays for vitamin D, iron and inflammatory biomarkers by study site.

Site	25(OH)D	Ferritin	Hepcidin	sTfR	Iron	Transferrin	CRP	ACT
**Kilifi, Kenya**	Chemiluminescent microparticle immunoassay (Abbott Architect, USA)	Microparticle Enzyme Immunoassay (Abbott Architect, USA)	DRG Hepcidin 25 [bioactive] high sensitive ELISA (DRG International, USA)	Human sTfR ELISA (BioVendor, Czech Republic)	MULTIGENT iron calorimetric assay, Abbott Architect, USA	Chemiluminescent Microparticle Immunoassay (Abbott Architect, USA)	MULTIGENT CRP Vario assay, (Abbott Architect, USA)	Not measured
**Banfora, Burkina Faso**	Chemiluminescent microparticle immunoassay (Abbott Architect, USA)	Microparticle Enzyme Immunoassay (Abbott Architect, USA)	DRG Hepcidin 25 [bioactive] high sensitive ELISA (DRG International, USA)	Human sTfR ELISA (BioVendor, Czech Republic)	MULTIGENT iron calorimetric assay, Abbott Architect, USA	Chemiluminescent Microparticle Immunoassay (Abbott Architect, USA)	MULTIGENT CRP Vario assay, (Abbott Architect, USA)	Not measured
**Entebbe, Uganda**	Chemiluminescent microparticle immunoassay (Abbott Architect, USA)	Microparticle Enzyme Immunoassay (Abbott Architect, USA)	DRG Hepcidin 25 [bioactive] high sensitive ELISA (DRG International, USA)	Human sTfR ELISA (BioVendor, Czech Republic)	Not measured	Chemiluminescent Microparticle Immunoassay (Abbott Architect, USA)	MULTIGENT CRP Vario assay, (Abbott Architect, USA)	Not measured
**Soweto, South Africa**	Chemiluminescent microparticle immunoassay (Abbott Architect, USA)	Microparticle Enzyme Immunoassay (Abbott Architect, USA)	DRG Hepcidin 25 [bioactive] high sensitive ELISA (DRG International, USA)	Human sTfR ELISA (BioVendor, Czech Republic)	Not measured	Chemiluminescent Microparticle Immunoassay (Abbott Architect, USA)	MULTIGENT CRP Vario assay, (Abbott Architect, USA)	Not measured
**West Kiang, The Gambia**	Chemiluminescent microparticle immunoassay (Abbott Architect, USA)	Microparticle Enzyme Immunoassay (Abbott Architect, USA)	Hepcidin-25 [human] Enzyme Immunoassay Kit (Bachem, Switzerland)	Quantikine sTfR ELISA kit, (R&D Systems, USA)	Ferrozine based photometry and colorimetry analyser (Hitachi 911, Tokyo, Japan)	Not measured	Not measured	Immunoturbidimetry Cobas Mira Plus Bioanalyser, Roche

Abbreviations: ACT: α1-antichymotrypsin; CRP: C-reactive protein; ELISA: enzyme-linked immunosorbent assay; and sTfR: soluble transferrin receptor. Hemoglobin levels for Ugandan children were adjusted for an altitude of greater than 1 km above sea level by subtracting 0.2 g/dL [52]. Iron measurements were not available for Ugandan and South African cohorts because their plasma samples were stored in ethylenediaminetetraacetic acid (EDTA) which chelates iron. Hepcidin values in Gambian samples were harmonized by first converting to the old DRG hepcidin assay values and thereafter to the new highly sensitive DRG hepcidin assay values [53].

Table S1. Effects of vitamin D status on iron deficiency, iron deficiency anemia and anemia in African children.

		Vitamin D status categories based on 25(OH)D levels						
		>75 nmol/L (reference)	50–75 nmol/L			<50 nmol/L		
**Proportion, *n*/total (%)**		2485/4509 (55.1%)	1674/4509 (37.1%)			350/4509 (7.8%)		
		n	***n***	**OR (95% CI)**	***p***	***n***	**OR (95% CI)**	***p***
**Iron deficiency**[1]	Overall[4]	2425/4399	1632/4399	1.12 (0.97, 1.30)	0.13	342/4399	1.98 (1.52, 2.58)	<0.0001
	Kenya	794/1322	450/1322	0.86 (0.65, 1.13)	0.27	78/1322	0.94 (0.53, 1.67)	0.84
	Uganda	725/1240	452/1240	0.95 (0.73, 1.23)	0.68	63/1240	1.84 (1.07, 3.15)	0.027
	Burkina Faso	178/319	122/319	1.12 (0.66, 1.90)	0.67	19/319	0.95 (0.31, 2.89)	0.93
	The Gambia	265/629	302/629	0.82 (0.54, 1.24)	0.35	62/629	1.06 (0.53, 2.13)	0.86
	South Africa	463/889	306/889	2.45 (1.78, 3.37)	<0.0001	120/889	4.80 (3.07, 7.52)	<0.0001
**Iron deficiency anemia**[2]	Overall[4]	1540/2880	1139/2880	0.94 (0.76, 1.15)	0.52	201/2880	1.42 (0.96, 2.10)	0.075
	Kenya	420/771	293/771	0.91 (0.61, 1.38)	0.68	58/771	0.96 (0.41, 2.26)	0.93
	Uganda	690/1182	430//1182	0.91 (0.67, 1.25)	0.57	62//1182	1.85 (1.00, 3.45)	0.052
	Burkina Faso	167/304	118/304	1.01 (0.57, 1.76)	0.98	19/304	0.77 (0.24, 2.53)	0.67
	The Gambia	263/623	298/623	0.98 (0.62, 1.57)	0.96	62/623	1.66 (0.81, 3.42)	0.17
	South Africa	n/a	n/a	n/a	n/a	n/a	n/a	n/a
**Anemia**[3]	Overall[4]	1587/2971	1175/2971	1.03 (0.87, 1.23)	0.73	209/2971	1.27 (0.90, 1.79)	0.17
	Kenya	430/793	301/793	0.65 (0.45, 0.93)	0.019	65/793	0.71 (0.38, 1.32)	0.28
	Uganda	719/1241	457/1241	1.08 (0.84, 1.39)	0.55	65/1241	1.08 (0.62, 1.87)	0.79
	Burkina Faso	175/314	119/314	1.37 (0.65, 2.88)	0.41	20/314	2.14 (0.26, 17.66)	0.48
	The Gambia	263/623	298/623	1.35 (0.96, 1.91)	0.086	62/623	2.75 (1.45, 5.17)	0.002
	South Africa	n/a	n/a	n/a	–	n/a	n/a	–

Abbreviations: 25(OH)D, 25-hydroxyvitamin D; n/a, not available. Odds ratios and *p* values were obtained from multivariable logistic regression analyses adjusted for age, sex, season, inflammation and study site. 25(OH)D and CRP levels were ln-transformed to make them normally distributed. [1]Iron deficiency was defined as either plasma ferritin <12µg/L or <30µg/L in the presence of inflammation in children <5 years old, or <15µg/L or <70µg/L in the presence of inflammation in children ≥5 years old; [2]iron deficiency anemia as the presence of both iron deficiency and anemia; [3]anemia was defined as hemoglobin <11 g/dL in children aged <5 years, or hemoglobin <11.5 g/dL in children ≥5 years; inflammation as CRP > 5 mg/L or ACT >0.6 g/L (ACT, but not CRP was available for The Gambia). The overall estimate was obtained by performing a meta-analysis of study site estimates using *metan* in STATA.

Table S2. Geometric means of biomarkers by country.

Biomarker means	Overall		Kenya		Uganda		Burkina Faso		The Gambia		South Africa[#]	
	n	Mean (95% CI)	*n*	Mean (95% CI)	*n*	Mean (95% CI)	*n*	Mean (95% CI)	*n*	Mean (95% CI)	n	Mean (95% CI)
25(OH)D (nmol/L)	4509	77.0 (76.3, 77.7)	1361	81.9 (80.4, 83.3)	1301	78.3 (77.0, 79.5)	329	77.3 (75.0, 79.6)	629	70.4 (69.0, 71.9)	889	72.8 (71.2, 74.5)
Ferritin (µg/L)	4399	20.2 (19.6, 20.8)	1322	21.3 (20.1, 22.5)	1240	20.7 (19.5, 21.9)	319	22.1 (19.6, 24.8)	629	25.3 (23.6, 27.0)	889	14.9 (14.0, 15.9)
Hepcidin (µg/L)	4308	6.4 (6.1, 6.6)	1255	5.6 (5.2, 6.0)	1266	6.8 (6.3, 7.2)	295	5.3 (4.5, 6.3)	619	6.1 (5.5, 6.9)	873	7.7 (7.1, 8.4)
sTfR (mg/L)	4379	10.0 (9.8, 10.2)	1344	18 (17.6, 18.4)	1274	6.8 (6.5, 7.1)	327	17.6 (16.6, 18.7)	546	3.4 (3.3, 3.5)	888	11.2 (10.9, 11.6)
Transferrin (µg/L)	3800	2.7 (2.7, 2.7)	1313	2.8 (2.7, 2.8)	1282	2.7 (2.6, 2.7)	316	2.7 (2.6, 2.8)	n/a	n/a	889	2.7 (2.7, 2.8)
Iron (µmol/L)	1664	6.9 (6.7, 7.0)	1337	6.4 (6.1, 6.6)	n/a	n/a	327	6.1 (5.7, 6.5)	n/a	8.6 (8.3, 8.9)	n/a	n/a
TSAT (%)	1612	10.1 (9.8, 10.4)	1297	9.2 (8.8, 9.6)	n/a	n/a	315	9.0 (8.3, 9.7)	n/a	12.8 (12.3, 13.4)	n/a	n/a
Hemoglobin (g/dL)	2971	10.4 (10.4, 10.5)	793	10.1 (10.0, 10.2)	1241	11 (10.9, 11.1)	314	9.5 (9.4, 9.6)	623	10.6 (10.4, 10.7)	n/a	n/a

Table S3. Effect of vitamin D status on individual measures of iron status.

Biomarker	*n*	Overall	Vitamin D status categories						
			>75 nmol/L (reference)	50–75 nmol/L			<50 nmol/L		
		Mean[1] (95% CI)	Mean (95% CI)	Mean (95% CI)	Beta (95% CI)[2]	*P* value	Mean (95% CI)	Beta (95% CI)[1]	*P* value
Ferritin (µg/L)	4358	20.2 (19.6, 20.8)	20.5 (19.6, 21.3)	21.0 (20.0, 22.0)	-0.05 (-0.11, 0.01)	0.10	15.2 (13.4, 17.2)	-0.35 (-0.45, -0.24)	<0.0001
Hepcidin (µg/L)	4265	6.4 (6.1, 6.6)	6.4 (6.1, 6.7)	6.6 (6.2, 7.1)	0.0002 (-0.08, 0.08)	1.0	5.3 (4.6, 6.1)	-0.30 (-0.45, -0.16)	<0.0001
sTfR (mg/L)	3791	10.0 (9.8, 10.2)	10.6 (10.4, 11.1)	9.2 (8.8, 9.5)	-0.05 (-0.08, -0.01)	0.013	9.0 (8.3, 9.8)	-0.08 (-0.14, -0.01)	0.029
Transferrin (µg/L)	3770	2.7 (2.7, 2.7)	2.8 (2.8, 2.8)	2.6 (2.6, 2.7)	-0.16 (-0.20, -0.12)	<0.0001	2.3 (2.2, 2.4)	-0.39 (-0.46, -0.32)	<0.0001
Iron (µmol/L)	1650	6.9 (6.7, 7.0)	6.4 (6.2, 6.7)	7.2 (7.0, 7.5)	0.03 (-0.02, 0.09)	0.25	8.4 (7.6, 9.2)	0.13 (0.02, 0.23)	0.015
TSAT (%)	1599	10.1 (9.8, 10.4)	9.2 (8.8, 9.5)	10.8 (10.3, 11.3)	0.05 (-0.01, 0.11)	0.082	13.5 (12.1, 15.0)	0.21 (0.09, 0.32)	0.0004
Hemoglobin (g/dL)	2939	10.4 (10.4, 10.5)	10.4 (10.3, 10.4)	10.5 (10.4, 10.6)	-0.04 (-0.14, 0.07)	0.50	10.4 (10.2, 10.6)	-0.22 (-0.42, -0.03)	0.025

Table S4. Interaction between 25(OH)D concentrations and inflammation and malaria in predicting measures of iron status.

Biomarker	*n*	Beta (95% CI)	*P value*	Beta (95% CI)	*P value*
**A. Inflammation[1]**		**Main effects (log vitamin D)**		**Interaction (log vitamin D x inflammation)**	
Log ferritin (μg/L)	4076	0.38 (0.28, 0.48)	<0.0001	-0.31 (-0.52, -0.10)	0.004
Log hepcidin (μg/L)	4019	0.22 (0.08, 0.36)	0.002	-0.19 (-0.47, 0.09)	0.19
Log sTfR (mg/L)	3512	0.09 (0.03, 0.16)	0.004	-0.04 (-0.17, 0.08)	0.51
Transferrin (μg/L)	3474	0.46 (0.39, 0.53)	<0.0001	-0.07 (-0.20, 0.06)	0.29
Log iron (μmol/L)	1359	-0.13 (-0.23, -0.03)	0.01	0.02 (-0.16, 0.21)	0.82
Log TSAT (%)	1307	-0.22 (-0.33, -0.11)	<0.0001	-0.01 (-0.22, 0.20)	0.95
Hemoglobin (g/dL)	2848	0.11 (-0.08, 0.29)	0.27	-0.02 (-0.39, 0.35)	0.92
**B. Malaria[2]**		**Main effects (log vitamin D)**		**Interaction (log vitamin D x malaria)**	
Log ferritin (μg/L)	3187	0.23 (0.01, 0.35)	<0.0001	0.001 (-0.33, 0.33)	1.00
Log hepcidin (μg/L)	3146	-0.003 (-0.17, 0.16)	0.98	-0.08 (-0.52, 0.35)	0.71
Log sTfR (mg/L)	2624	0.22 (0.14, 0.30)	<0.0001	-0.04 (-0.25, 0.17)	0.68
Transferrin (μg/L)	2585	0.41 (0.33, 0.49)	<0.0001	0.16 (-0.05, 0.36)	0.13
Log iron (μmol/L)	1359	-0.22 (-0.32, -0.11)	<0.0001	0.15 (-0.10, 0.41)	0.24
Log TSAT (%)	1307	-0.31 (-0.42, -0.19)	<0.0001	0.11 (-0.18, 0.39)	0.47
Hemoglobin (g/dL)	2848	-0.01 (-0.19, 0.17)	0.90	-0.004 (-0.47, 0.46)	0.99

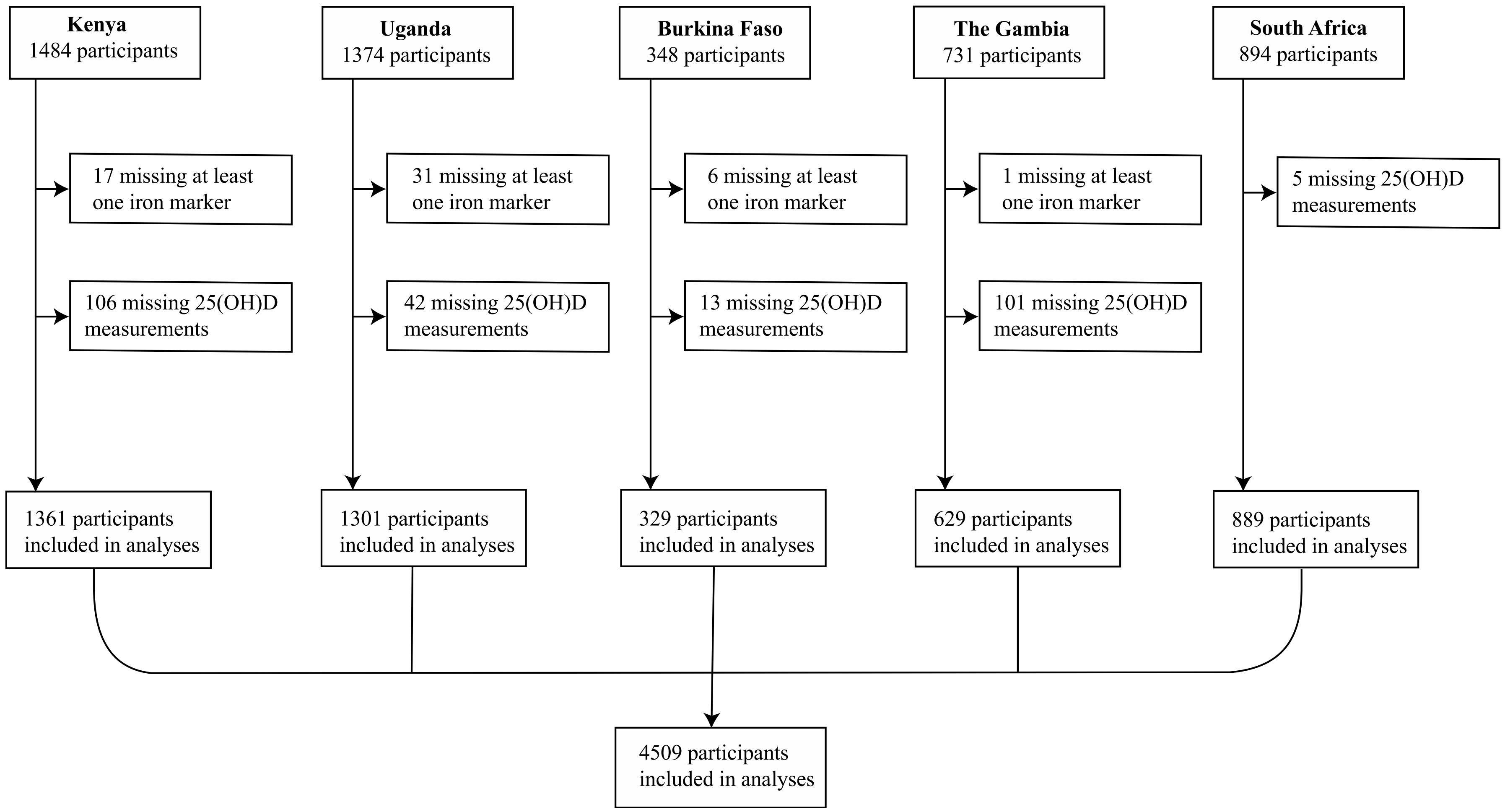

Figure S1. Flow chart of study participants. Iron status and 25(OH)D concentrations were measured on available stored samples from study cohorts across Africa (as described in the Methods). Study participants that did not have at least one marker of iron status or 25(OH)D concentrations measured were excluded from the analyses.

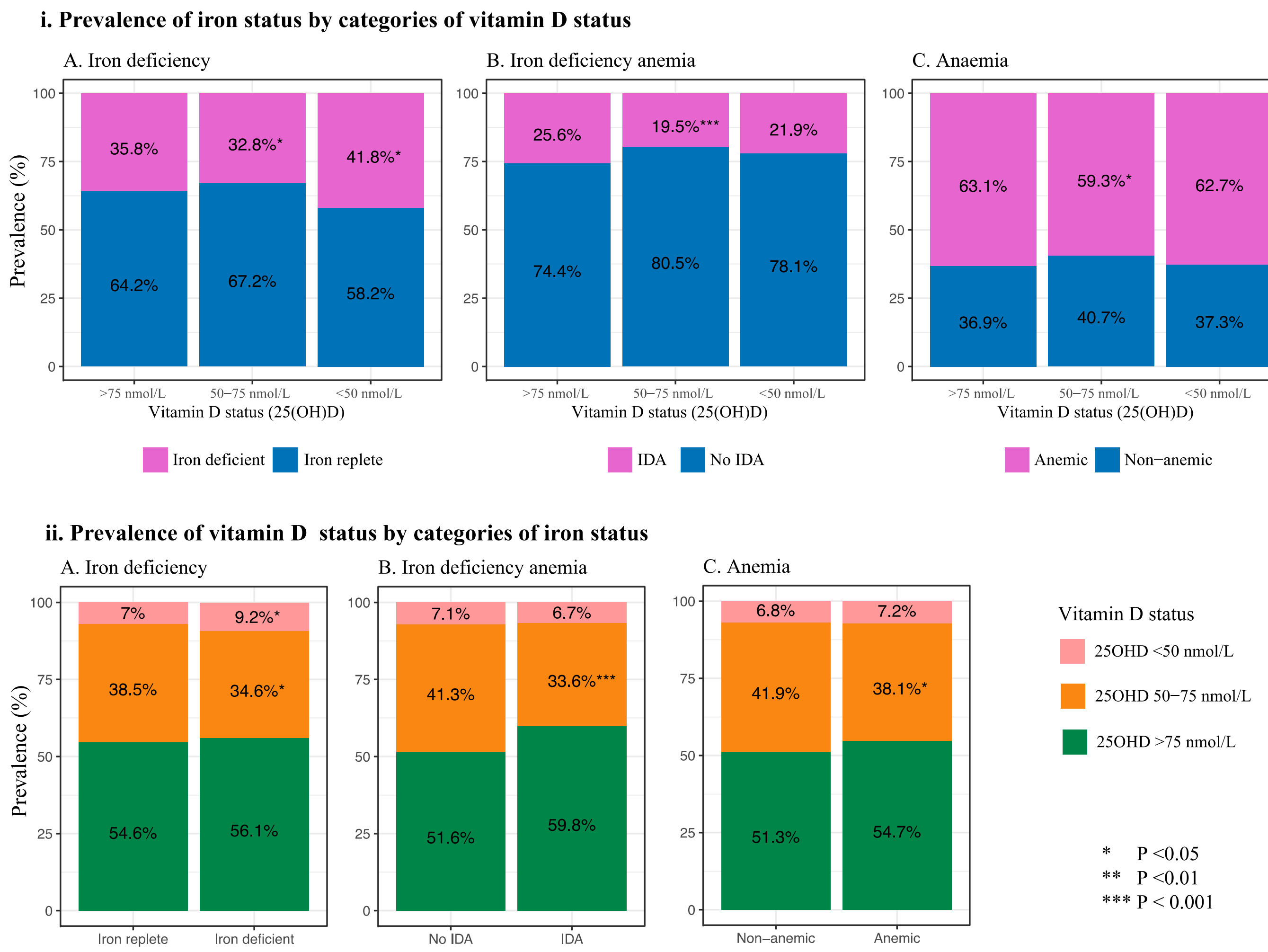

Figure S2. Prevalence of iron status by categories of vitamin D status (i) and prevalence of vitamin D status by categories of iron status (ii) in African children. A chi-squared test (*prtest)* was used to test the significance in the difference in proportion of low vitamin status (25(OH)D concentrations <50 or 50–75 nmol/L) within each category with the first category as the reference.

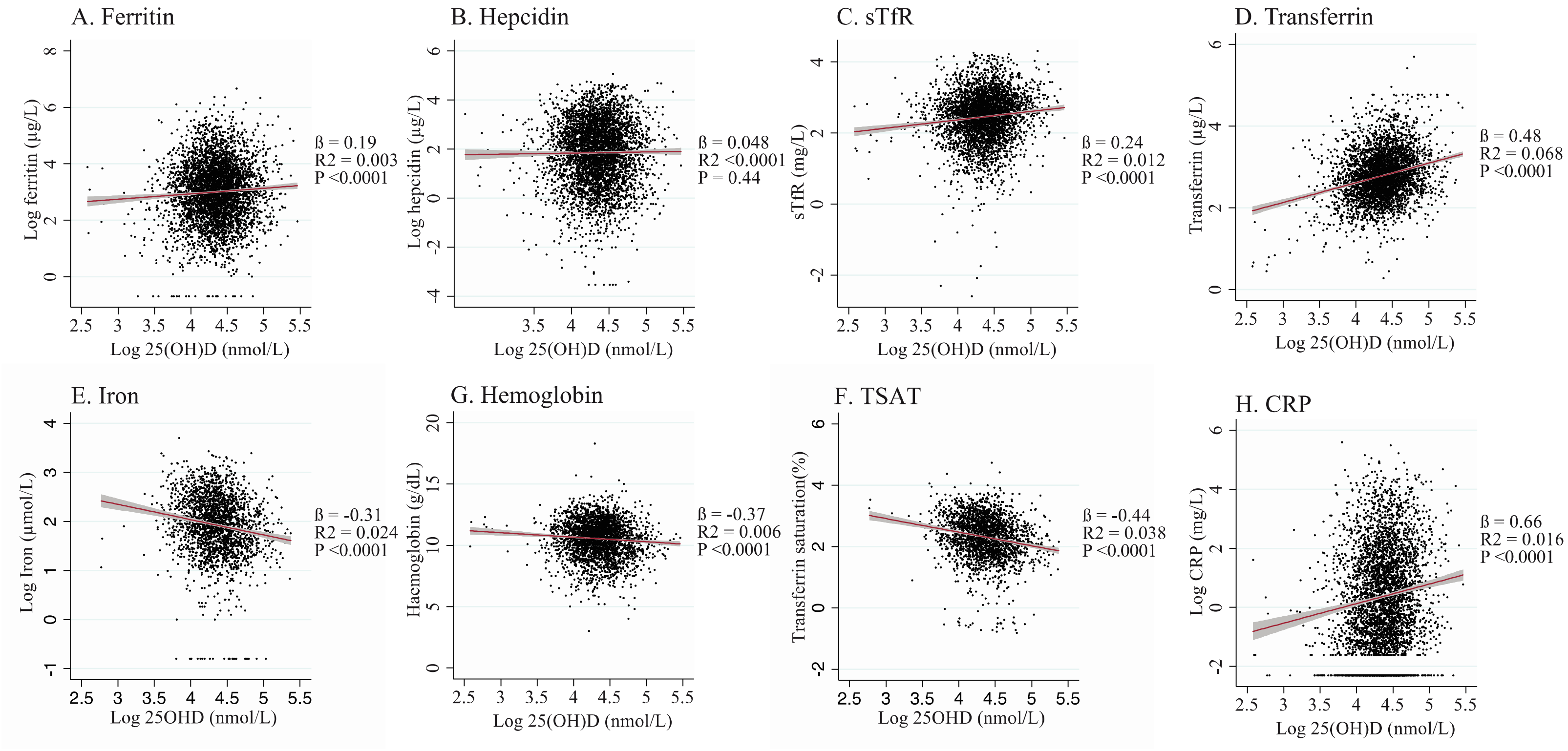

Figure S3. Scatter and regression plots of markers of iron status and C-reactive protein against log 25(OH)D concentrations. Abbreviations: TSAT, transferrin saturation; sTfR, soluble transferrin receptor; CRP, C-reactive protein.

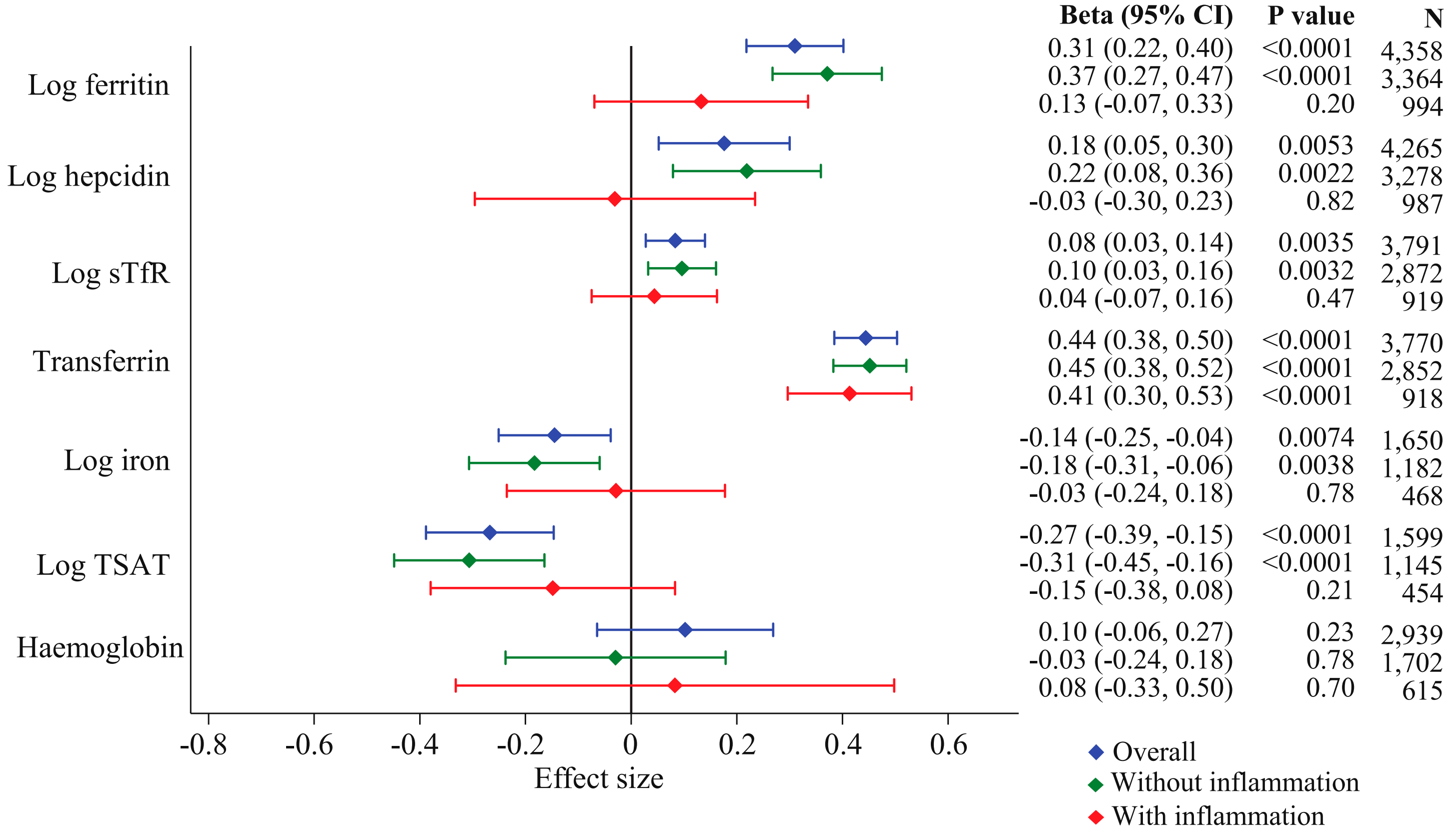

Figure S4. Association between 25(OH)D concentrations and iron markers overall (dark grey) and in African children with and without inflammation (black and light grey lines respectively). Both the dependent and independent variables (except transferrin and hemoglobin) were log-transformed before inclusion in the linear model. Thus, the beta values may be interpreted as follows: a 1% change in 25(OH)D concentrations corresponds to a beta % change in the individual iron markers. All models were adjusted for age, sex, season, and study site and the overall model was additionally adjusted for inflammation. Error bars indicate 95% confidence intervals. Abbreviations: TSAT, transferrin saturation; sTfR, soluble transferrin receptor.

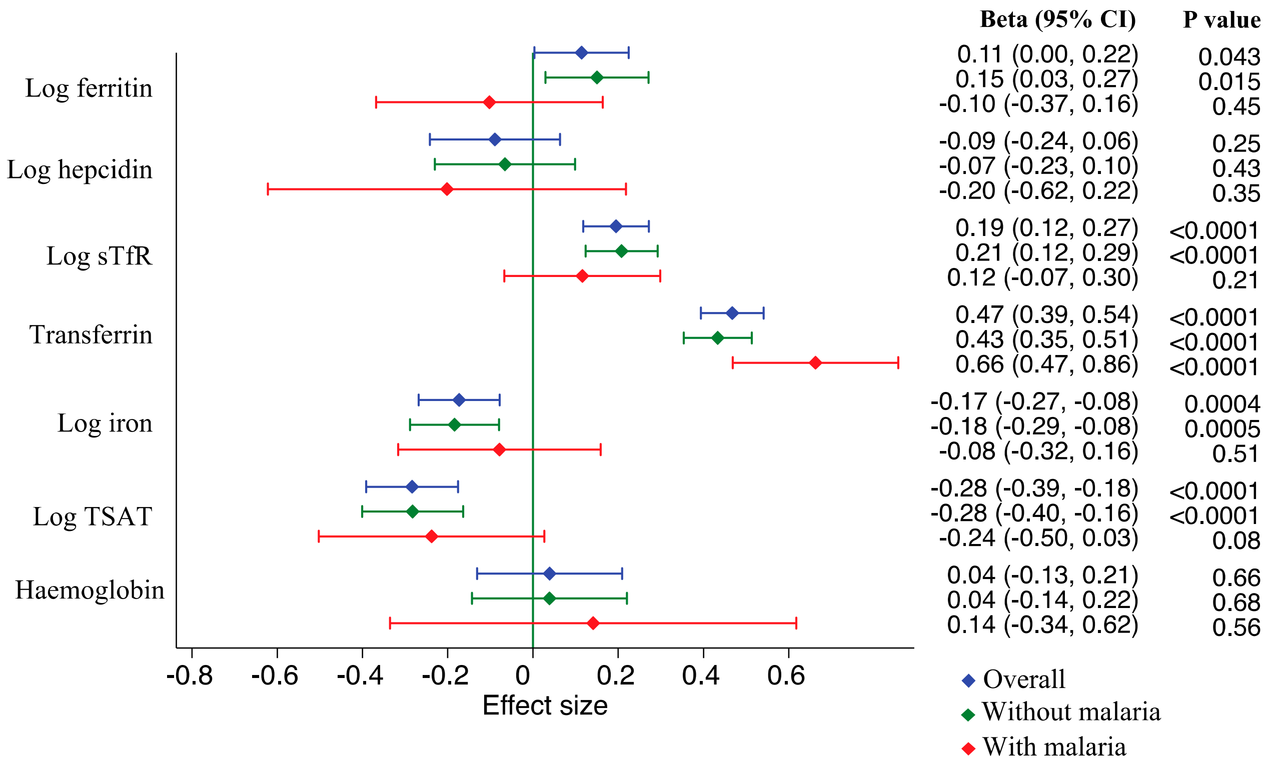

Figure S5. Association between 25(OH)D concentrations and iron markers overall (blue) and in children with and without malaria in Africa (red and green lines, respectively). Both the dependent and independent variables (except transferrin and hemoglobin) were log-transformed before inclusion in the linear model. Thus, the beta values may be interpreted as follows: a 1% change in 25(OH)D concentrations corresponds to a beta % change in the individual iron markers. All models were adjusted for age, sex, season, study site and inflammation and the overall model was additionally adjusted for malaria. South African children were not included in the analyses since they were not exposed to malaria. Error bars indicate 95% confidence intervals. Abbreviations: TSAT, transferrin saturation; sTfR, soluble transferrin receptor.

Supplemental References

53. World Health Organization. Haemoglobin concentrations for the diagnosis of anaemia and assessment of severity. **2011**.
54. Wray, K.; Allen, A.; Evans, E.; Fisher, C.; Premawardhena, A.; Perera, L.; Rodrigo, R.; Goonathilaka, G.; Ramees, L.; Webster, C., et al. Hepcidin detects iron deficiency in Sri Lankan adolescents with a high burden of hemoglobinopathy: A diagnostic test accuracy study. *Am. J. Hematol.* **2017**, *92*, 196-203, doi:10.1002/ajh.24617.
